# Our Trip to Salt Spring

**Published:** 1886-09-20

**Authors:** 


### OUR TRIP TO SALT SPRING.

Since the last issue of the RECORD our Managing Editor did himself the pleasure to take a brief season of recreation at the above named Spring, which is situated in Douglas County, Ga., 20 miles West of Atlanta, in the immediate vicinity of Salt Spring Station on the Georgia Pacific Railroad.

Salt Spring Station is the point especially selected for disembarking for the Spring. There is here a hotel, hastily but neatly constructed, containing about fifty rooms. The place is eligibly located, being high, dry, and healthful, and is destined, as we believe, to be rapidly built up, and to constitute a place of great resort for health and pleasure in the future. Mr. Marsh, the present proprietor of the Spring, has, we learn, already contracted for the erection of another large hotel in the grove, about two hundred yards from the present building. It is to be a three-story structure, 250 feet front, with two wings running back 175 feet each. Water is to be brought from the Spring in pipes, and the grounds are to be variously improved and ornamented, for the pleasure, health and comfort of visitors.

From the hotel to the Spring a narrow-gauge road has been constructed, and neat passenger coaches, to convey visitors to and from the Spring—only about a mile distant. Along the route of this narrow-gauge road the country is being cleaned up, with a view to the erection of dwellings. There are on this line many beautiful situations for cottages, which will doubtless be bought up and settled at no distant day.

In approaching the Spring, there is a gentle decline in the grade of the road. The Spring is situated in the center of a glade, which is said at one time to have been densely covered with a thick and tangled growth of trees, vines and cane-brake, such as are often seen in the branch-bottoms throughout the beautiful sections of Middle and Upper Georgia. The variety of trees and native shrubbery was, at this spot, doubtless very great originally, for, though the undergrowth has been to a large extent cleaned off for some distance around, there is yet to be found a great number and variety of beautiful trees in the groves about the Spring; among these, we noticed all kinds of oak, the hickory, the pine, black gum, sweet gum, the elm, the red haw, the black haw, the hackberry, the dogwood, the hazel bush, the wild cherry, the live oak, the redbud, etc. A singular and characteristic feature exists in respect to the immediate growth at and below the Spring for a considerable distance—it is the moss which is seen upon the trees. This moss is peculiar, having a rich grayish-green color. It is unlike the moss of the low country, being of somewhat shorter fiber and of finer and more beautiful texture. So far as we can learn there is no such moss elsewhere to be found in all this section of country—a fact which, taken in connection with the fact that it extends to only a short distance below the Spring, where the earth may be supposed to be saturated with the mineral substances of the water, shows that the growth of the moss is caused by the water, and that this water is peculiar and different in this particular from all other waters known.

The glade extends a considerable distance above and below the Spring ; a beautiful little babbling brook runs along its center, and numerous granite bowlders, varying in size and shape—some of them as much as twenty or thirty feet in diameter—are scattered promiscuously in the vicinity of the Spring, and along the narrow-gauge railway. It is a singular fact that most of these bowlders, while sufficiently slant on one side to permit the pedestrian to reach their top, have a bluff or perpendicular side, presenting, in this respect, so many miniature Stone Mountains. These natural advantages and peculiarities give to the surroundings a wild and rugged aspect as romantic as it is beautiful.

Of the Spring itself little is known previous to the year 1883, although certain of the older citizens tell of climbing the trees near the Spring, forty or fifty years ago, to shoot the deer—as large numbers of these animals frequented the Spring to drink the water and lick the salt that was contained in the adjacent soil. During the war, a Mr. Denmead and a Mr. Johnson attempted to make salt by evaporating the water. In this they failed, as the per centage of salt in the water is but small. It is said that, in clearing away the mud and enlarging the Spring, they came, at the depth of about three feet, to a rock which had been scooped out like a basin, the size of a half-barrel, as a recepticle for the water. The basin had been carved out, in a neat and somewhat artistic manner, long years before, doubtless by the Indians who occupied the country as late as 1825. Among the natives known to the pioneers of that time was a chief having the euphoneous name of Sweet Water. His wigwam was on the creek near this Spring. The creek has now the name of Sweet Water. Whether the Indian was named for the creek or the creek for the Indian, or whether the Spring may not have furnished the name for both the Indian and the creek we did not ascertain.

It is related by persons residing in the vicinity that the parties who cleaned out the Spring in 1883 found beneath the mud, in the excavation made by the salt-makers, the head of a beef, slaughtered by them in 1864, which was fresh and untainted, although it had remained in the Spring a period of seventeen years ! This is mentioned in proof of the antiseptic properties of the water, which from that time began to attract special attention for its medicinal properties.

Dr. Bogan, the present resident physician at the Springs, first visited the place in July, 1883. His health was at the time almost hopelessly impaired. He soon recovered. The fame of the Spring, from that time, rapidly increased, so much so that large numbers from different sections of the country, and variously afflicted, flocked to the Spring, and were almost uniformly cured of their diseases. Since then, the reputation of the Spring has rapidly extended, and the visitors have constantly increased until, during the recent Summer season, thousands have visited the Spring, and the waters are being extensively shipped and sold. All this has resulted without newspaper advertising, the remarkable merits of the water being only proclaimed by those who have been cured or benefited thereby. The proprietor did not advertise the water, because he had not developed the property, and there was not yet sufficient accommodation provided for the people. He hopes by next season to have ample room for all, and to greatly improve and beautify the grounds, and to add largely to the attractions and beauties of the place.

For the analysis of the water the reader is referred to the advertisement on another page of this journal. The intelligent physician will at once perceive, in the minerals contained in this water, a rare and important therapeutical combination. It is alkaline, alterative, antibilious, tonic, gently diuretic, deobstruent, and nervine—properties which adapt it to a wide application in the treatment of disease. We were told by the resident physician that its effects may be varied by the greater or less quantity used, and by taking it cold or hot. Many find that a small quantity taken hot on going to bed acts as a laxative, producing bilious discharges. Locally applied for the sting of insects it gives instantaneous relief. In acid dyspepsia it acts admirably ; in many kidney affections it has proved a superior remedy ; in rheumatism, when taken in connection with bathing, it has been found very useful. In skin diseases it is pronounced invaluable, the resident physician affirming positively that he has never known it to fail in a single instance, when perseveringly used. In scrofulous maladies it is also highly lauded.

Many improvements are contemplated by Mr. Marsh for the next season. Already there are neat and well-arranged bathing-rooms, and over the Spring there is a large and elegant pavilion, 44 x 48 feet in dimension, with marble floor and comfortable seats ; here numerous visitors are almost constantly found engaged in social conversation, drinking the healing waters, and not infrequently

"Tripping the light fantastic toe"

to the strains of delightful music.

